# Tumor apelin and obesity are associated with reduced neoadjuvant chemotherapy response in a cohort of breast cancer patients

**DOI:** 10.1038/s41598-021-89385-z

**Published:** 2021-05-10

**Authors:** Florian Gourgue, Françoise Derouane, Cedric van Marcke, Elodie Villar, Helene Dano, Lieven Desmet, Caroline Bouzin, Francois P. Duhoux, Patrice D. Cani, Bénédicte F. Jordan

**Affiliations:** 1 Biomedical Magnetic Resonance Research Group, UCLouvain, Louvain Drug Research Institute, Université catholique de Louvain, Brussels, Belgium; 2 Metabolism and Nutrition Research Group, Louvain Drug Research Institute, WELBIO (Walloon Excellence in Life Sciences and BIOtechnology), UCLouvain, Université catholique de Louvain, Brussels, Belgium; 3 Department of Medical Oncology, Institut Roi Albert II, Cliniques universitaires Saint-Luc and Institut de Recherche Expérimentale et Clinique, UCLouvain, Brussels, Belgium; 4 Breast Clinic, Institut Roi Albert II, Cliniques Universitaires Saint-Luc, Brussels, Belgium; 5 Department of Pathology, Cliniques Universitaires St Luc, Brussels, Belgium; 6 Statistical Methodology and Computing Service, LIDAM, Université Catholique de Louvain, Brussels, Belgium; 7 Imaging Platform 2IP, Institut de Recherche Expérimentale et Clinique (IREC), UCLouvain, Université catholique de Louvain, Brussels, Belgium

**Keywords:** Breast cancer, Cancer therapy, Prognostic markers

## Abstract

Obesity is a known factor increasing the risk of developing breast cancer and reducing disease free survival. In addition to these well-documented effects, recent studies have shown that obesity is also affecting response to chemotherapy. Among the multiple dysregulations associated with obesity, increased level of the apelin adipokine has been recently shown to be directly involved in the association between obesity and increased breast cancer progression. In this study, we analyzed in a retrospective cohort of 62 breast cancer patients the impact of obesity and tumoral apelin expression on response to neoadjuvant chemotherapy. In the multivariate logistic regression, obesity and high tumoral apelin expression were associated with a reduced response to NAC in our cohort. However, obesity and high tumoral apelin expression were not correlated, suggesting that those two parameters could be independently associated with reduced NAC response. These findings should be confirmed in independent cohorts.

## Introduction

Breast cancer (BC) is the most common cancer and the leading cause of cancer death among women^1^. It is now recognized that obesity, a condition that has reached pandemic proportions, is a risk factor for BC^2^. This association is especially well described for postmenopausal luminal BC but has also been observed for other BC subtypes regardless of the menopausal status^3–6^. Several potential mechanisms linking obesity and cancer have been identified as for instance higher estrogen exposure^7,8^, systemic low-grade inflammation^9,10^, increased insulin and insulin-like growth factor expression^11,12^ or altered adipokines secretion^13,14^. Obese patients have increased circulating levels of apelin^15,16^. Upon binding to the apelin receptor APJ, this adipokine is involved in several physiological functions as angiogenesis, heart contractility, energy metabolism and tumor progression^17,18^. Moreover, we recently demonstrated in an vivo study that this adipokine is implicated in the relation between obesity and BC^19^. Reproducing obesity-related levels of apelin is sufficient to promote BC growth and metastatization^19^. Besides promoting BC, recent data showed that obesity affects response to neoadjuvant or adjuvant chemotherapy for BC patients^20–22^. As tumor apelin expression or its receptor APJ have been associated with poor survival in humans^23–26^, we hypothesized that the apelinergic system could also be implicated in the adverse relation between obesity and pathological complete response (pCR) to neoadjuvant chemotherapy (NAC) in BC patients.

## Results

### Patients’ characteristics

**Figure 1:**
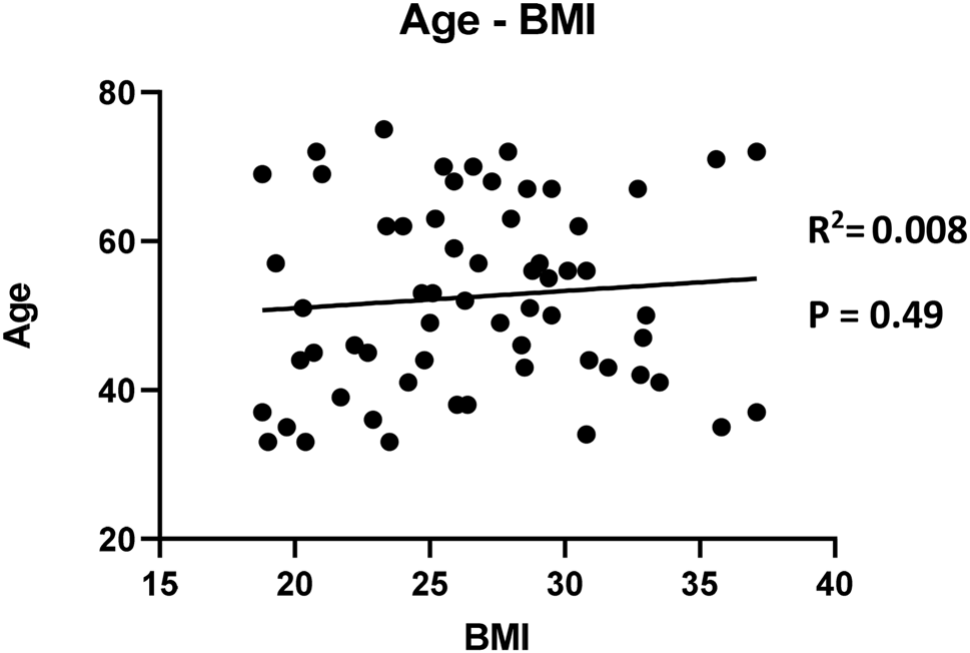
Correlation between age and BMI of patients (N = 62).

Body mass index (BMI) at diagnosis was used to classify 62 non-metastatic BC patients into three categories: normal weight (BMI < 25 kg/m^2^), overweight (BMI 25–30 kg/m^2^) and obese (BMI > 30 kg/m^2^) (Table 1). Thirty-five percent of patients were of normal weight, 40% were overweight and 25% were obese. The mean age of patients at diagnosis was 52.6 years old with a standard deviation of ± 12.4 (range 33–75). There was a trend towards overrepresentation of older patients in the overweight group (56.8 ± 9.96) as compared to the normal weight group (49.1 ± 13.73). This tendency was not observed in the obese category (50.5 ± 12.86). Altogether, age did not correlate with BMI in this cohort (Fig. 1). Compared to the normal weight and obese patients, overweight patients were more likely postmenopausal (41–40–72% of patients, respectively, p = 0.01). Two obese patients (3%) were diabetic. The majority of BC cases in the normal weight and obese groups were luminal. BC subtype was not significantly different between the subgroups. However, the overweight BC group was numerically enriched in triple negative BC cases, compared to normal weight and obese patients (36–18–7% respectively). Tumor size and cell proliferation were not significantly different among the three BMI categories. The majority of BC were of grade III. Nevertheless, tumor grade significantly diverged between the three subgroups, with a lower proportion of high-grade tumors in the obese patients (60%). Node infiltration did not differ between the subgroups.

**Table 1:** Clinical characteristics of patients based on BMI category (N = 62).

	Normal weight (BMI 18.5–24.9)	Overweight (BMI 25–29.9)	Obese (BMI > 30)	Total	p-value
	N (%)	N (%)	N (%)	N (%)	
BMI repartition	22 (35%)	25 (40%)	15 (25%)	62 (100%)	
Mean age (year) ± SD	49.1 (± 13.73)	56.8 (± 9.96)	50.5 (± 12.86)	52.6 (± 12.43)	0.16
**Menopausal status**					
Premenopausal	13 (59%)	7 (28%)	9 (60%)	29 (47%)	0.01
Postmenopausal	9 (41%)	18 (72%)	6 (40%)	33 (53%)	
**Type II diabetic**					
Yes	0 (0%)	0 (0%)	2 (13%)	2 (3%)	*
No	22 (100%)	25 (100%)	13 (87%)	60 (97%)	
**Molecular subtype**					
HR + HER2−	9 (41%)	7 (28%)	10 (66%)	26 (42%)	
HR + HER2+	5 (23%)	5 (20%)	1 (7%)	11 (18%)	0.1
HER2+	4 (18%)	4 (16%)	3 (20%)	11 (18%)	8
TNBC	4 (18%)	9 (36%)	1 (7%)	14 (22%)	
**High tumor size (> 2 cm)**					
Yes	17 (77%)	18 (72%)	8 (53%)	43 (69%)	0.28
No	5 (23%)	7 (28%)	7 (47%)	19 (31%)	
**Tumor grade**					
I–II	1 (5%)	6 (24%)	6 (40%)	13 (21%)	0.03
III	21 (95%)	19 (76%)	9 (60%)	49 (79%)	
**High Ki67 (> 15%)**					
Yes	20 (91%)	23 (92%)	15 (100%)	58 (94%)	0.49
No	2 (9%)	2 (8%)	0 (0%)	4 (6%)	
**Nodal infiltration**					
Yes	14 (64%)	16 (64%)	12 (80%)	42 (68%)	0.51
No	8 (36%)	9 (36%)	3 (20%)	20 (32%)	
**pCR**					
Yes	12 (55%)	11 (44%)	2 (13%)	25 (40%)	0.03
No	10 (45%)	14 (56%)	13 (87%)	37 (60%)	

*BMI* Body-mass index, *SD* standard deviation, *pCR* pathological complete response, *HR* hormone receptor, *TNBC* triple negative breast cancer. Anova one-way analysis for mean age, Chi square test for other parameters. P-values are annotated.

*Too few parameters to perform a Chi-square analysis.

The pCR rate was significantly different between the three subgroups, with a trend towards decreased response to chemotherapy with increasing BMI category (p = 0.03).

### BMI and tumor apelin are associated with NAC pCR in BC

**Figure 2:**
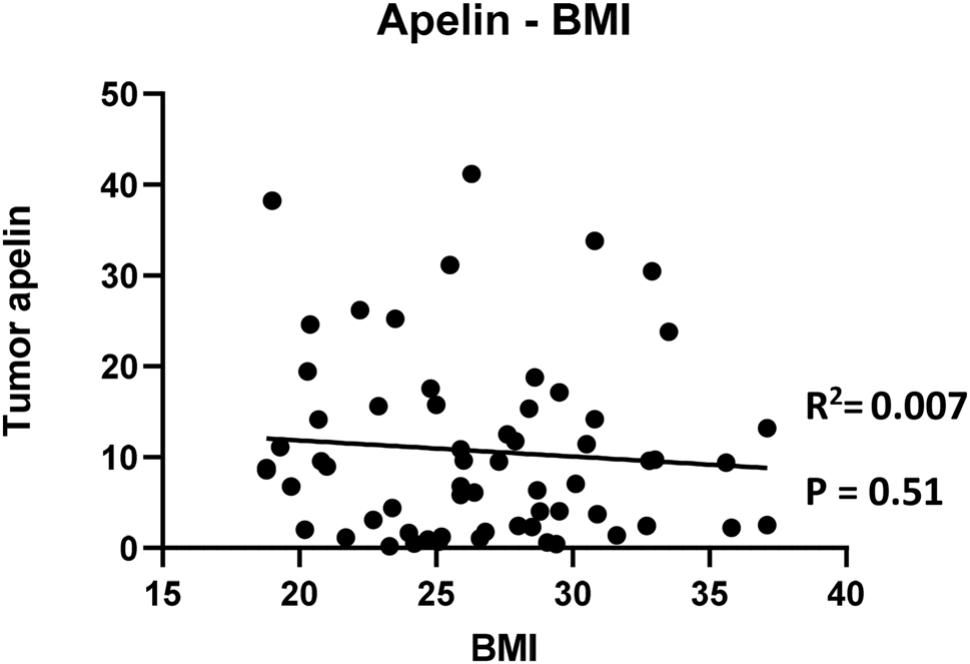
Correlation between tumor apelin expression and BMI of patients (N = 62).

We investigated the link between pCR after NAC and BMI by univariate and multivariate logistic regression, accounting for several clinical relevant parameters [BMI, menopausal status, tumor grade, tumor size, nodal involvement and hormone receptor expression (Table 2)]. As our team recently highlighted in mouse models that obesity promotes tumor apelin expression and that high circulating apelin favors BC aggressiveness^19^, we also analyzed tumoral expression of the adipokine apelin and its receptor and tested their association with pCR by univariate logistic regression and in the multivariate logistic regression model. Interestingly, only BMI [Odds ratio (OR) of 0.86, 95% confidence interval (CI) 0.74–0.99] and tumor apelin expression (OR of 0.90, 95% CI 0.83–0.97) were significantly associated with pCR in the multivariate analysis. No other factor was significantly associated with pCR. However, tumor apelin did not correlate with BMI (Fig. 2).

**Table 2:** Univariate and multivariate logistic regression of clinical factors and odds ratio of pathological complete response (N = 62).

	Univariate analysis		Multivariate analysis	
	Odds ratio (95% CI)	p-value	Odds ratio (95% CI)	p-value
BMI	0.88 (0.78–0.99)	**0.03**	0.86 (0.74–0.99)	**0.04**
Apelin tumoral	0.95 (0.88–1.00)	**0.06**	0.90 (0.83–0.97)	**0.01**
APJ tumoral	1.02 (0.96–1.07)	0.59	1.04 (0.98–1.13)	0.22
Postmenopausal	1.21 (0.44–3.39)	0.72	0.85 (0.23–2.98)	0.80
High grade (III)	2.72 (0.73–13.2)	0.14	2.49 (0.47–15.67)	0.30
Size > 2 cm	1.71 (0.56–5.67)	0.35	1.52 (0.36–6.73)	0.57
Node	1.02 (0.35–3.10)	0.97	1.73 (0.42–7.76)	0.46
HR +	0.59 (0.21–1.66)	0.31	0.51 (0.13–1.90)	0.32

*BMI* Body mass index, *HR+ * hormone receptor positive, *APJ* apelin receptor**.**

## Discussion

In this study, we retrospectively assessed the response to anthracycline and taxanes-based NAC in 62 early BC patients. As already highlighted in previous studies^20^, obesity was in our cohort significantly associated with a poorer response to NAC. We also observed numerical but not statistically significant trends towards higher pCR rate in high-grade tumors, and lower pCR rate in luminal tumors.

To further explore this association between weight status and pCR rate, we measured the tumoral expression of apelin and APJ. Interestingly, we discovered that high tumor apelin expression was associated with a lower rate of pCR in these patients. This finding further suggests that this adipokine plays a detrimental role in cancer. Indeed, several preclinical and clinical studies have shown that apelin correlates with metastatization and poor overall survival^23,27^. Here, our clinical exploratory study suggests that tumor expression of apelin in BC is also associated with a poor response to NAC, a factor associated with worse disease-free survival^28^.

In our previous preclinical study, we found that BC tumors developing in obese mice display an increased tumoral apelin expression. In the current cohort, BMI and tumor apelin did not correlate, suggesting these two parameters might independently affect response to NAC. This should be interpreted cautiously, as only 3% of patients were diabetic in our cohort, whereas the mice we used in preclinical studies were diabetic. Indeed, insulin is the main inducer of apelin expression^29^ and could explain why obese subjects have increased apelin expression levels.

The small sample size and retrospective nature of our study are other limitations. Indeed, we could question the representativeness of our cohort as no factor commonly associated with pCR shows a significant association with this parameter in our study. Therefore, our findings are only hypothesis generating and must be validated in an independent, prospective cohort. In addition, a new study would allow us to refine our model by including other obesity-related parameters such as insulin sensitivity and circulating levels of apelin expression. Indeed, in a retrospective study, circulating levels of apelin were directly correlated with cancer stage in several different forms of tumors, including BC^30^. Moreover, even if the use of BMI as a parameter for obesity is used in daily clinical practice, the use of waist-to-hip ratio as parameter for central obesity could be more appropriate to study the implication of obesity and apelin in response to NAC in BC patients. Larger studies would also allow to reduce heterogeneity between patients by considering separately the different BC subtypes, but also menopausal status.

In conclusion, in this retrospective exploratory study on 62 early BC patients treated with taxane and anthracycline-based NAC, obesity and high tumor apelin expression were independently associated with poorer pCR rates. This observation supports the notion that besides their role in the development of BC, both obesity and specific adipokines could play a role in the response to chemotherapy. Confirmation of these findings in independent cohorts would allow assessing their clinical relevance.

## Patients and methods

### Patients

We retrospectively collected a series of 64 patients with early BC, treated with NAC at Cliniques universitaires Saint-Luc (a tertiary care center in Brussels, Belgium) between 2012 and 2020. Patients had received similar chemotherapy regimen and had remaining pre-treatment tumor samples available. Two anorexic patients with a BMI below 18.5 were excluded and the final cohort was 62 patients. Baseline information at diagnosis included anthropometric measurements, menopausal and diabetic status. This study was approved by the Ethics Committee of Cliniques universitaires Saint-Luc (2017/25JUL/376). All research was performed in accordance with relevant guidelines/regulations, and informed consent was obtained from all participants.

### Tumors biopsies

Patients underwent biopsy at diagnosis. Hormone receptor status [estrogen receptor (ER) and progesterone receptor (PR)] was evaluated by immunohistochemistry (IHC) and reported with the Allred score. Human epidermal growth factor receptor 2 (HER2) gene amplification status was determined by IHC and considered as positive for a staining superior to 10% (3+). In case of ambiguous IHC score (2+), FISH was performed to determine the *HER2* gene amplification. Subtypes are categorized as following: hormone receptor positive (HR + HER2−) for tumor positive for either ER and/or PR and HER2 negative, HR + HER2+ for tumor positive for ER and/or PR and HER2 positive, HER2+ for tumor positive for HER2 and triple negative in case of negativity of ER, PR and HER2. Histological grade was assessed by the Nottingham scoring system. High Ki-67 index was determined for IHC staining above 15%^31^. Nodal invasion at diagnosis suspected by clinical evaluation or ultrasound had to be confirmed by cytopunction. Pathological complete response (pCR) was achieved if no invasive carcinoma was found in the breast and in the axilla (ypT0/is ypN0).

### Neoadjuvant regimen

Patients underwent neoadjuvant regimen including combination of anthracycline and cyclophosphamide followed by taxanes. Depending on the recommendations at the time of diagnosis, patients have received either: 4 cycles of 5-fluorouracile (500 mg/m^2^), epirubicin (100 mg/m^2^), cyclophosphamide (500 mg/m^2^) followed by 4 cycles of docetaxel (100 mg/m^2^) or 4 cycles of epirubicin (90 mg/m^2^) and cyclophosphamide (600 mg/m^2^) followed by 12 cycles of paclitaxel (80 mg/m^2^). Trastuzumab (6 mg/m^2^) was administered if case of HER2 positive status, and in one case, the patient also received Pertuzumab (fixed dose 420 mg). Three patients received carboplatin (AUC 6) in association of taxanes because of non-response to anthracyclines regimen. In some cases, patients did not receive the complete anthracycline regimen because of intolerance, or the complete taxol regimen because of neuropathy (Supplementary Table 1).

### Immunohistochemistry

Biopsies were fixed in 4% paraformaldehyde for 24 h at room temperature before processing for paraffin embedding. Sections of 5 µm were submitted to endogenous peroxidases inhibition. Sections were then subjected to antigen retrieval in 10 mM citrate buffer pH 5.7 and to blocking of aspecific antigen binding sites (TBS containing 5% BSA and 0.05% Triton). Anti-apelin and anti-APJ primary antibody (Apelin: Abcam59469, APJ: Abcam214369) were incubated in TBS containing 1% BSA and 0.05% Triton and detected with anti-rabbit horseradish peroxidase-conjugated polymer secondary antibodies (Agilent) overnight at 4 °C. HRP was then visualized by DAB (Agilent). Cell nuclei were counterstained with hematoxylin. Stained slides were then digitalized using a SCN400 slide scanner (Leica Biosystems) at 40 × magnification and tumor area were detected by a Pathologist. Percentage of stained tissue was analyzed using Visiopharm software.

### Statistical methods

Statistical analyses were performed using Graphpad Prism 8.0. For descriptive analyses, categorical parameters were presented as distribution of frequencies and continuous parameters as mean ± standard deviation. Descriptive analyses were performed using Chi-square test for categorical parameters and one-way Anova for continuous parameters. Factor associations with pathological complete response were tested by univariate and multivariate logistic regression. A p-value of ≤ 0.05 was considered significant.

## Supplementary Information

Supplementary Information.
