# Supplementary material for: Tumor apelin and obesity are associated with reduced neoadjuvant chemotherapy response in a cohort of breast cancer patients
Source: Sci Rep. 2021 May 10;11:9922. doi: 10.1038/s41598-021-89385-z (PMC8110990; doi:10.1038/s41598-021-89385-z)

**Tumor apelin and obesity are associated with reduced neoadjuvant chemotherapy response in a cohort of breast cancer patients**

Florian Gourgue^1-2^, Françoise Derouane^3^, Cedric van Marcke^3^, Elodie Villar^4^, Helene Dano^5^, Lieven Desmet^6^, Caroline Bouzin^7^, Francois P. Duhoux^3^*, Patrice D. Cani^2^*, Bénédicte F. Jordan^1^*

**1** Biomedical Magnetic Resonance Research Group, UCLouvain, Louvain Drug Research Institute, Université catholique de Louvain, Brussels, Belgium

**2** Metabolism & Nutrition Research Group, Louvain Drug Research Institute, WELBIO (Walloon Excellence in Life sciences and BIOtechnology), UCLouvain, Université catholique de Louvain, Brussels, Belgium

**3** Department of Medical Oncology, Institut Roi Albert II, Cliniques universitaires Saint-Luc and Institut de Recherche Expérimentale et Clinique, UCLouvain, Brussels, Belgium

**4** Breast Clinic, Institut Roi Albert II, Cliniques universitaires Saint-Luc, Brussels, Belgium

**5** Cliniques Universitaires St Luc, Departement of Pathology, Brussels, Belgium

**6** Statistical Methodology and Computing Service, LIDAM, Université catholique de Louvain, Brussels, Belgium

**7** Imaging platform 2IP, Institut de Recherche Expérimentale et Clinique (IREC), UCLouvain, Université catholique de Louvain, Brussels, Belgium

**Corresponding authors (*):**

Benedicte F. Jordan benedicte.jordan@uclouvain.be

Patrice D. Cani patrice.cani@uclouvain.be

François P. Duhoux francois.duhoux@uclouvain.be

Supplementary data

**Supplementary table 1: individual chemotherapy regimen (N=62).**


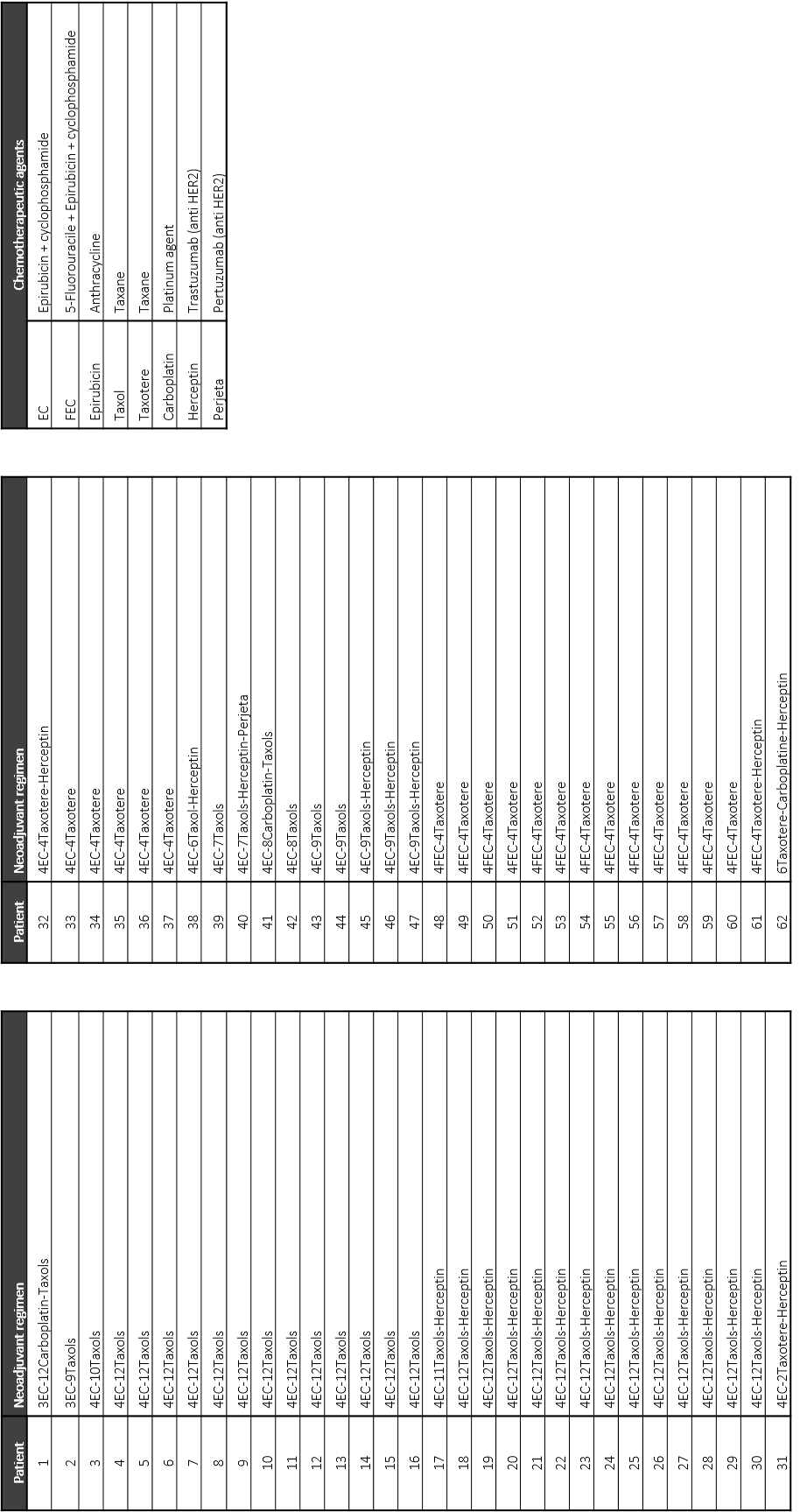

Supplement: Supplementary file 1 — Supplementary Information. [file 41598_2021_89385_MOESM1_ESM.docx]
